# Acknowledgment to the Reviewers of *Medicines* in 2022

**DOI:** 10.3390/medicines10020014

**Published:** 2023-01-18

**Authors:** Medicines Editorial Office

**Affiliations:** MDPI AG, St. Alban-Anlage 66, 4052 Basel, Switzerland

High-quality academic publishing is built on rigorous peer review. *Medicines* was able to uphold its high standards for published papers due to the outstanding efforts of our reviewers. Thanks to the efforts of our reviewers in 2022, the median time to first decision was 21 days and the median time to publication was 50 days. Regardless of whether the articles they examined were ultimately published, the editors would like to express their appreciation and thank the following reviewers for the time and dedication that they have shown *Medicines*:
Aaron PinkhasovMaja Rogić VidakovićAbraham TsitlakidisMangesh HadeÁdám SchléglManlio SantilliAdina SantanaMariagiovanna CantoneAdrian BuzeaMariana BarbosaAgnieszka WareńczakMary Angela O'NealAlberto CaggiatiMassimo MaccagnoAldo BoveMatteo SangiorgiAlessandro MaugeriMaziar OveiseeAmanda ChamiehMichel MarreAnand ParamasivamMichèle M. IskandarAngel Josabad Alonso-CastroMohamad Nurul IslamAnkit TanwarMohamed El RaeyAnnalisa GrandiMohammad Mahbubur Rahman Khan MamunAntonio BulumMohammad ZulkifliAshlie BernhiselMohind C. MohanAtoshi BanerjeeMunenori UemuraCarmine IzzoNadia HussainCaroline LangNicolae GicaChang-Hoon ChoiNusima MohamedChristiane GerardNwabundo AnusimChristina E. KostaraOlga SysoevaChu John M. T.Omid MirmosayyebClarice ChoongOrli Friedman-EldarCraig R. BaileyOurlad Alzeus TantengcoCristian PersuPaez-Escamilla A. ManuelCristina BuiguesPatrick BurnsCristina Martin-SabrosoPetca Razvan-CosminCyrus MotamedPierre Le BarsDania Olivia Govea-AlonsoPradeep Kumar SahuDanijela TasicPrzemysław DomaszewskiDavid AchudhanRaymond J. WallsDavid SinclairRobert FlisiakDenis MalaiseRoberta TittarelliDiana MostafaRoberto ColluDieter BettelheimRojek SebastianDimitri KakabadseRosa Maria GerardiDivanshu ShuklaSabrina MartinezEdinson Dante Meregildo RodriguezSafa FarajElisa Lopez-VarelaSamuele CerutiElsebeth LyngeSanjay KumarEman ThabetSara MoraisEnas M. HefzySarvesh SabarathinamEssam Al-MoraissiShahzad IrfanEva ZetterbergSharon MichelhaughEvren AydoğmuşShatavisha DasguptaFabio MiraldiSigmund KharaschFeng HeSimran TandonFlemming BjerrumSlava BergerGiovanni VolpicelliSlaven Lupi-FerandinGiuseppina MieleStefania ZanettiGuillermo Moreno-SanzStylianos TzedakisHadia AbdelaalSulaiman S. IbrahimHani Nasser AbdelhamidSumedha BaggaHeather ZwickeySummaiya ZareenHidetaka HamasakiTadashi KawaiHumaira JamshedTarun KeswaniIlya KlabukovThomas LamInmaculada López-IzquierdoTrang NguyenJamir Pitton RissardoUnaib RabbaniJean Claude De MauroyValentina StranieroJoel EllwangerVincenzo CozzolinoJosé Manuel Montes-RodríguezWachira WongtanasarasinJuan YangWenjuan WangJulien BogousslavskyXiaohu HuangKana PuspitaYangtian YiKarthikeyan SubbarayanYoonJung LeeLara S. McClureYu-Chi LeeLaura SyronYu-Chiang WangLiana TodorYuhki SatoLisethe MeijerYurang ParkLorena Caridad Y. López Del RíoZeinab MajidMade SuandikaZoubir Djerada


